# Timing is everything: a collection on how clocks affect resilience in biological systems

**DOI:** 10.12688/f1000research.5756.1

**Published:** 2014-11-13

**Authors:** Ilia N. Karatsoreos, Bruce S. McEwen

**Affiliations:** 1 Department of Integrative Physiology and Neuroscience, Washington State University, Pullman, WA, 99164, USA; 2 Harold and Margeret Milliken Hatch Laboratory of Neuroendocrinology, The Rockefeller University, New York, NY, 10065, USA

## Abstract

Why do some people get sick, and others do not? This basic question, of vulnerability and resilience, is of crucial importance in physical and mental health research. Determining factors that impart resilience, or contribute to vulnerability, could be a turning point in our fundamental understanding of disease. This collection explores current data on the role of two systems that seem indispensible for health: Circadian rhythms, and Sleep. While we have learned a great deal about the mechanisms that control these two biological processes, we are only at the beginning of our understanding of how these systems impact mood, cognition, immune function, and metabolism. The concepts of resilience and vulnerability may be useful in understanding the myriad effects of circadian rhythm and sleep disruption on mental and physical health, and perhaps provide new insights to how we can predict – and protect against – negative outcomes while amplifying positive health effects.

## Editorial

Why do some people get sick, and others do not, even when they are exposed to the same pathogen? This basic question, of vulnerability and resilience in physical health, is also of crucial importance in the study of mental health. For instance, why of two individuals who experience exposure to the same traumatic event, does only one develop Post Traumatic Stress Disorder (PTSD)?

Some individuals show exceptional resilience while others show remarkable vulnerability to environmental challenges, and the differences in the outcomes between these groups could not be starker. Being able to unravel the basic mystery of resilience of physiological systems and behavioral processes promises to revolutionize health research and health care. By understanding the mechanisms that contribute to resilience, this could illuminate methods to stimulate those same mechanisms in others, potentially protecting against many diseases. Similarly, by understanding the pathways contributing to vulnerability, interventions could be devised to mitigate those risk factors. Determining the factors that impart resilience, or contribute to vulnerability, could be a turning point in our understanding of mental and physical disease.

The purpose of this collection is to explore the role of two separable yet intimately intertwined systems in resilience and vulnerability: Circadian rhythms and sleep. These processes are crucial for health, but we still know little about the specific mechanisms that mediate their effects on organismal function and dysfunction. This is especially important considering that shift work, jet lag, and artificial light at night have become commonplace in our modern industrialized society.

Circadian (daily) rhythms allow organisms, and even individual cells, to maintain homeostasis in a constantly changing world. These rhythms allow both temporal compartmentalization of incompatible processes within a cell, and a mechanism to predict regular changes in the environment. The wealth of genetic information on the function of circadian clocks in many tissues has provided tools to dissect the mechanisms that regulate timing on the molecular, cellular, circuit, and whole organism levels. However, we still lack a fundamental understanding of how disruptions of the clock lead to the negative mental and physical health effects observed when circadian rhythms are dysregulated.

Perhaps the most salient circadian rhythm in humans is the daily alternation between sleep and wake, and while timing of sleep is modulated on a circadian basis, sleep is regulated by several distributed systems throughout the brain. While we think of sleep as occurring on the whole organismal level, sleep processes occur even at the neural circuit level. We have gained significant insight into the mechanisms that regulate sleep, but the functions of sleep still remain a deep mystery. Moreover, the processes by which disruptions in sleep affect diverse processes from emotionality, learning and memory, to immune function and metabolism, remain unknown.

Both circadian rhythms and sleep seem indispensible for mental and physical health. We hope this collection will provide stimulating and timely discussion on the roles of circadian rhythms and sleep as gatekeepers of resilience and vulnerability.

